# The Synthesis and Anion Recognition Property of Symmetrical Chemosensors Involving Thiourea Groups: Theory and Experiments

**DOI:** 10.3390/s151128166

**Published:** 2015-11-06

**Authors:** Xuefang Shang, Zhenhua Yang, Jiajia Fu, Peipei Zhao, Xiufang Xu

**Affiliations:** 1 Department of Chemistry, Xinxiang Medical University, Xinxiang 453003, China; E-Mail: YY851@xxmu.edu.cn; 2 School of Pharmacy, Xinxiang Medical University, Xinxiang 453003, China; E-Mails: 15736991800@163.com (J.F.); 15738671962@163.com (P.Z.); 3 Department of Chemistry, Nankai University, Tianjin 300071, China; E-Mail: xxfang@nankai.edu.cn

**Keywords:** thiourea derivative, symmetrical structure, chemosensor, theoretical investigation

## Abstract

The synthesis of four symmetrical compounds containing urea/thiourea and anthracene/nitrobenzene groups was optimized. *N,N’*-Di((anthracen-9-yl)-methylene)thio-carbonohydrazide showed sensitive and selective binding ability for acetate ion among the studied anions. The presence of other competitive anions including F^−^, H_2_PO_4_^−^, Cl^−^, Br^−^ and I^−^ did not interfere with the strong binding ability. The mechanism of the host-guest interaction was through multiple hydrogen bonds due to the conformational complementarity and higher basicity. A theoretical investigation explained that intra-molecular hydrogen bonds existed in the compound which could strengthen the anion binding ability. In addition, molecular frontier orbitals in molecular interplay were introduced in order to explain the red-shift phenomenon in the host-guest interaction process. Compounds based on thiourea and anthracene derivatives can thus be used as a chemosensor for detecting acetate ion in environmental and pharmaceutical samples.

## 1. Introduction

The artificial design and synthesis of biologically important anion chemosensors has been attracting considerable attention in the field of supramolecular chemistry because of the important roles of various anions in biological, chemical and environmental systems [1,2,3,4,5,6,7,8,9,10,11,12]. In various important anion analytes, carboxylate ions are important products of metabolic processes in the human body [13,14,15,16]. Therefore, the acetate ion and the carboxylate biomolecules may be more important than other biological functions anions. So far, the focus has been mainly on the design a chemosensor which has the ability to bind the biologically important anions. The binding sites in artificial chemosensors are significant and often include amide [17], urea [18], hydroxyl [19,20] and pyrrole [21] moieties due to the strong binding ability to act as hydrogen-bond donors. Among the possible hydrogen-bond donors, thiourea derivatives are particularly good [22,23,24]. Anthracene and nitrobenzene were chosen as the fluorogen and chromophore [25,26] because they can act as an optically sensitive indicator for anion binding ability especially for the detection of acetate anion.

**Scheme 1:**
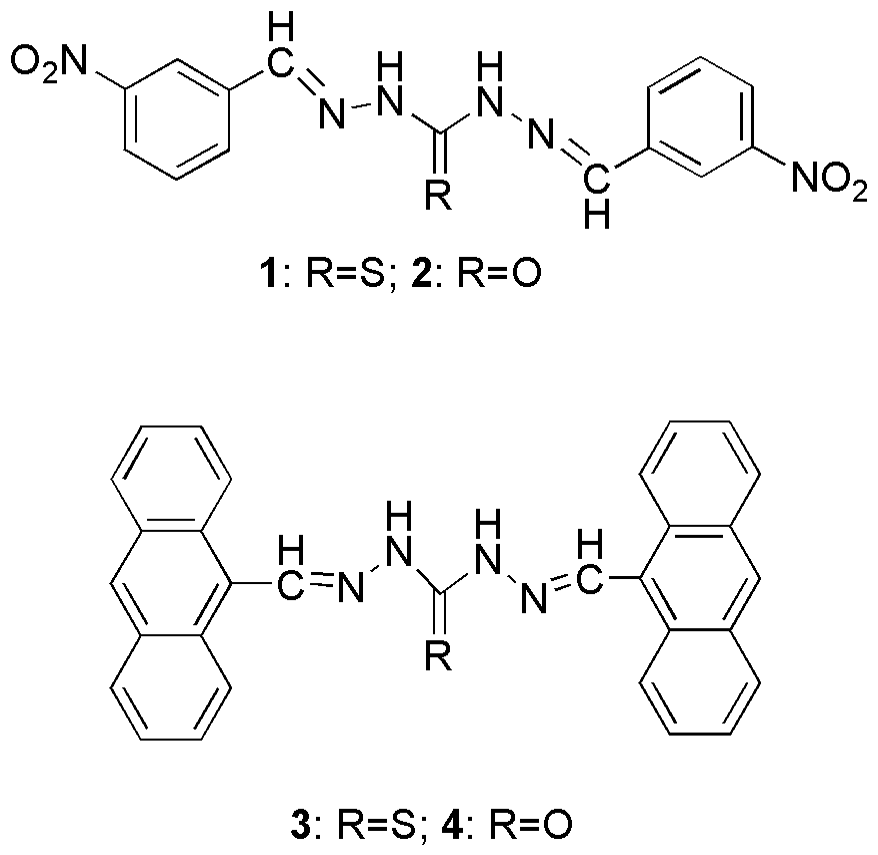
The synthesized compounds.

Bearing in mind the above considerations, we report in this paper the synthesis of some symmetrical chemosensors based on thiourea derivatives (Scheme 1) and their anion binding ability. The obtained experimental and theoretical results showed that compound **3** with an anthracene framework could effectively recognize and sense AcO^−^ in DMSO.

## 2. Experimental Section

### 2.1. General Information 

All the reagents and solvents used for the experiments were purchased commercially in the purest form available and were used without further purification. All the anions, in the form of the corresponding tetrabutylammonium salts [such as (*n*-C_4_H_9_)_4_NF, (*n*-C_4_H_9_)_4_NCl, (*n*-C_4_H_9_)_4_NBr, (*n*-C_4_H_9_)_4_NI, (*n*-C_4_H_9_)_4_NAcO, (*n*-C_4_H_9_)_4_NH_2_PO_4_], were purchased from Sigma-Aldrich Chemical Co. (Shanghai, China), and stored in a desiccator under vacuum. Tetra-*n*-butylammonium salts were dried for 24 h in vacuum with P_2_O_5_ at 333 K. Dimethyl sulfoxide (DMSO) was distilled under vacuum after drying with CaH_2_. C, H, N elemental analyses were performed on a Vario-EL instrument (Heraeus, Frankfurt, Germany). ^1^H-NMR spectra were recorded on a Varian UNITY Plus-400 MHz Spectrometer (Bruker, Karlsruhe, Germany). ESI-MS was performed with a LC-MS apparatus (Agilent, Palo Alto, CA, USA). UV-Vis titration experiments were done on a Shimadzu UV2550 spectrophotometer (Shimadzu, Kyoto, Japan) at 298 K. Fluorometric titration was performed on a Cary Eclipse Fluorescence spectrophotometer (Agilent, Palo Alto, CA, USA) at 298 K. The binding constant, *K*_s_, was obtained by non-linear least squares calculation method for data fitting. In order to investigate the anion binding behaviours of the receptor, theoretical calculations were carried out with the Gaussian03 computer program. Optimizations of the receptor and anions have been carried out without symmetry constraints by applying B3LYP/3-21G method. 

### 2.2. Chemistry

The four target compounds (**1**, **2**, **3** and **4**) were synthesized by the condensation of 3-nitrobenzalhehyde or 9-anthracenecarbaldehyde with diamineurea (thiourea).

*N,N’*-Di(3-nitrophenylmethylene)thiocarbonohydrazide (1): 3-Nitrobenzaldehyde (1 mmol, 151 mg), diaminethiourea (2 mmol, 212 mg) and three drops of acetic acid were dissolved in ethanol (35 mL) and then the resulting solution was refluxed for 2 h under N_2_. The reaction mixture was cooled to room temperature after the reaction. The precipitate formed was filtered, washed twice with ethanol (2 × 5 mL) and a yellow solid was obtained. Yield: 87%. ^1^H-NMR (DMSO-*d_6_*): δ 12.00 (s, 2H, NH), 10.74 (s, 2H, phenyl-H), 8.79 (s, 2H, CH), 8.28–8.19 (m, 4H, phenyl-H), 7.72–7.69 (t, 2H, phenyl-H). Elemental analysis: Calc. for C_15_H_12_N_6_O_4_S: C, 48.38; H, 3.25; N, 22.57; Found: C, 48.51; H, 3.02; N, 22.79. ESI-MS (*m/z*): 371.2 (*M*-H)^−^.

*N,N’*-Di(3-nitrophenylmethylene)carbonohydrazide (2): This compound was synthesized according to the synthesis method of compound **1** with the replacement of diaminourea for diaminothiourea and a white solid was obtained. Yield: 79%. ^1^H-NMR (DMSO-*d_6_*): δ 11.09 (s, 2H, NH), 8.58 (s, 2H, CH), 8.22 (s, 2H, phenyl-H), 8.21–8.17 (m, 4H, phenyl-H), 7.74–7.70 (t, 2H, phenyl-H). Elemental analysis: Calc. for C_15_H_12_N_6_O_5_: C, 50.57; H, 3.39; N, 23.59; Found: C, 50.89; H, 2.97; N, 23.66. ESI-MS (*m/z*): 355.0 (*M*-H) ^−^.

*N,N’*-Di((nthracen-9-yl)-methylene)thiocarbonohydrazide (3): It was synthesized according to the synthesis method of compound **1** with the replacement of 9-anthracencarbaldehyde for 3-nitro-benzaldehyde and an orange solid was obtained. Yield: 82%. ^1^H-NMR (DMSO-*d_6_*): δ 12.23 (d, 2H, NH), 10.45 (d, 1H, anthracen-H), 9.21 (s, 1H, anthracen-H), 9.17 (s, 2H, CH), 8.75–8.62 (dd, 4H, anthracen-H), 8.18–8.11 (m, 4H, anthracen-H), 7.65–7.54 (m, 8H, anthracen-H). Elemental analysis: Calc. for C_31_H_22_N_4_S: C, 77.15; H, 4.59; N, 11.61; Found: C, 76.86; H, 4.23; N, 11.78. ESI-MS (*m/z*): 481.4 (*M*-H) ^−^.

*N,N’*-Di((anthracen-9-yl)-methylene) carbonohydrazide (4): It was synthesized according to the synthesis method of compound **3** with the replacement of diaminourea for diaminothiourea and a glassy yellow solid was obtained. Yield: 91%. ^1^H-NMR (DMSO-*d_6_*): δ 11.03 (d, 2H, NH), 9.11 (s, 2H, CH), 8.98 (d, 2H, anthracen-H), 8.73–8.61 (dd, 4H, anthracen-H), 8.21–8.19 (m, 4H, anthracen-H), 7.63–7.56 (m, 8H, anthracen-H). Elemental analysis: Calc. for C_31_H_22_N_4_O: C, 79.81; H, 4.75; N, 12.01; Found: C, 79.64; H, 4.92; N, 11.88. ESI-MS (*m/z*): 465.5 (*M*-H) ^−^.

## 3. Results and Discussion

### 3.1. UV-Vis Titration

**Figure 1:**
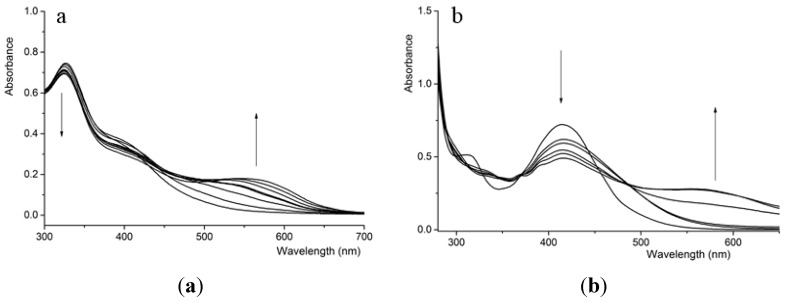
UV-Vis spectral changes of compounds (4.0 × 10^−^^5^ mol·L^−1^) in DMSO upon addition of AcO^−^ (0–50 equiv), (**a**) **1**; (**b**) **3**. The arrow indicates the direction of increase of acetate concentration.

The spectrophotometric titrations of four compounds with the various anions (AcO^−^, F^−^ H_2_PO_4_^−^, Cl^−^, Br^−^ and I^−^) have been determined in DMSO solution. The results indicated that the spectral responses of urea derivatives (**2** and **4**) were very weak with the stepwise addition of the various anions and so the host-guest interaction could be ignored. While, the remarkable spectral responses of thiourea derivatives (compound **1** and **3**) occurred after various anions were added (Figure 1). The absorption spectra of free **1** displayed an absorption peak centered at 325 nm with a weak shoulder peak at 400 nm, which could be assigned to the intramolecular charge-transfer transitions (ICT) of the thiourea moiety and the excitation of π electrons in the aromatic system, respectively [27,28]. After the AcO^−^ ion was added to the solution of compound **1**, the absorbance intensity at 325 nm was reduced. Simultaneously, a new absorbance band at 550 nm developed and the color of the solution changed from yellow to orange-red. Interestingly, the orange-red solution of complex **1-**AcO^−^ change back to the yellow of compound **1** due to the addition of a protic solvent (such as ethanol or H_2_O), which suggested the host-guest interaction was derived from hydrogen bonding in essence [29,30]. Particularly, the additions of F^−^ and H_2_PO_4_^−^ induced similar changes in UV-Vis spectra of **1** compared with AcO^−^, but the additions of excess equiv. of Cl^−^, Br^−^ and I^−^ ions resulted in weak changes in the UV-Vis spectra, which indicated compound **1** showed different binding ability with AcO^−^, F^−^, H_2_PO_4_^−^, and almost no or very weak binding ability with Cl^−^, Br^−^ and I^−^. 

The free compound **3** showed a strong absorption band at 420 nm and a weak absorption band at 320 nm which could be assigned to the intramolecular charge-transfer transitions of the thiourea moiety and the excitation of π electrons in the anthracene system, respectively. After the AcO^−^ ion was added, the absorption peak at 420 nm weakened gradually and a new absorption peak at 550 nm developed. On the other hand, the color of the solution changed from yellow to orange. The development of a new absorption peak and color changes could be contributed to the development of hydrogen bonds between compound **3** and the anion. The appearance of isosbectic points indicated the formation of a single complex species between compound **3** and AcO^−^. The addition of F^−^, H_2_PO_4_^−^ ions induced a similar spectral response and the addition of Cl^−^, Br^−^, I^−^ did not lead to any spectral changes of compound **3**.

### 3.2. Fluorescence Response

**Figure 2:**
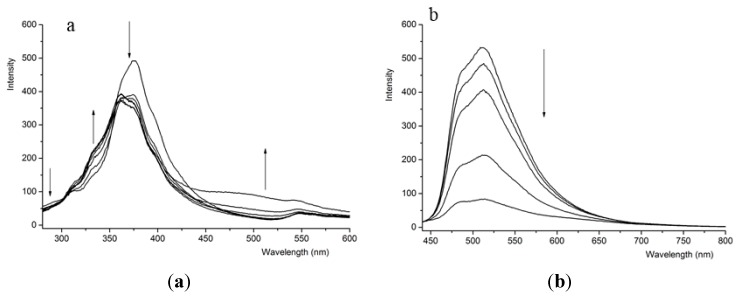
Fluorescence titration of compounds (4.0 × 10^−5^ mol·L^−1^) with AcO^−^ in DMSO at 298 K. (**a**) **1**; (**b**) **3**.

The anion binding behavior of two compounds (**1** and **3**) was also investigated by fluorescence response, which was consistent with the UV-Vis titrations (Figure 2). Compound **1** exhibited a strong emission band centered at 375 nm (λ_ex_ = 266 nm), probably due to the excited state proton transfer (ESIPT) process. ESIPT was a well-known process in intramolecular hydrogen bonded Schiff bases, where a fast tautomeric transformation from Schiff base to imine occurred in the excited state [31]. Upon the addition of AcO^−^, the emission intensity of compound **1** at 375 nm decreased (Figure 2). Addition of 50 equiv. of AcO^−^ resulted in a clear fluorescence enhancement at 500 nm. Importantly, the quenching of compound **1** induced by acetate anion was not affected by the presence of other competitive anions. Compared with AcO^−^ ion, there were similar changes in the fluorescence emission of **1** induced by the addition of F^−^ and H_2_PO_4_^−^ ions. Nevertheless, the fluorescence emission of compound **1** was insensitive to the additions of excess equiv. of Cl^−^, Br^−^ and I^−^ ions. 

Free compound **3** exhibited a strong emission centered at 512 nm upon excitation at 412 nm. Compared with compound **1**, a significant quenching was apparent in the emission intensity of compound **3** with the stepwise addition of AcO^−^. The fluorescence quenching was occurred due to the blocking of the ESIPT process on the NH groups of compound **1** with acetate ion through hydrogen bond followed by the intermolecular charge transfer (ICT). In addition, the presence of F^−^ and H_2_PO_4_^−^ induced similar changes in the fluorescence spectra with AcO^−^, but the additions of excess equiv. Cl^−^, Br^−^ and I^−^ ions induced only slight effects on the fluorescence intensity.

### 3.3. The Calculation of Binding Constant

The Job-plot analysis suggested the spectral changes could be ascribed to the formation of 1:1 host-guest complexes. The UV-Vis spectral data were used to calculate the binding constant (*K*_s_) between two compounds and anions by applying Equation (1):

*X* = *X*_0_ + 0.5 Δε {*c*_H_ + *c*_G_ + 1/*K*_s_ − [(*c*_H_ + *c*_G_ + 1/*K*_s_)^2^ – 4*c*_H_*c*_G_]^1/2^} (1)

where, *c*_G_ and *c*_H_ are the concentration of guest and host, respectively. *X* is the absorbance intensity measured with different concentrations of the anions. *X*_0_ is the absorbance intensity of the free compound. *K*_s_ is the binding constant for the host-guest complexation. Δε is the change in the molar extinction coefficient.

The binding constants listed in Table 1 were obtained using the non-linear least squares calculation method [32,33,34]. Obviously, the binding ability of two compounds (**1** and **3**) with various anions was in the order AcO^−^ > F^−^ > H_2_PO_4_^−^ >> Cl^−^, Br^−^, I^−^. The binding ability of acetate ion was the strongest among the anions tested which suggested multiple hydrogen-bonding was the key factor in the host-guest interaction. What’s more, the strong binding ability was also related with the host-guest matching degree. Acetate anion is a triangular shape and the O-C-O angle is about 120^°^, therefore the distance between two oxygen atoms might fit two hydrogen atoms on the interacted sites of two compounds **1** and **3** in a triangular configuration. According to literatures [35,36], the stronger the basicity of the anion is, the higher the binding ability with a receptor is. So the binding constant of F^−^ ion was higher than that of H_2_PO_4_^−^ ion due to the acidity sequence: F^−^ > H_2_PO_4_^−^. The binding ability of Cl^−^, Br^−^ and I^−^ was very weak and could be ignored due to the weak spectral responses. For the same anion, the anion binding ability of compound **3** was higher than that of compound **1**. The reason may be related with the strong conjugation effect of anthracene.

**Table 1:** The binding constants *K*_s_ (M^−1^) of the compounds with various anions.

Anion ^a^	Compound	
	1	3
AcO^−^	(1.33 ± 0.08) × 10^4^	(3.93 ± 0.06) × 10^4^
F^−^	(2.06 ± 0.14) × 10^3^	(2.64 ± 0.02) × 10^4^
H_2_PO_4_^−^	(9.25 ± 0.27) × 10^2^	(4.45 ± 0.24) × 10^3^
Cl^−^, Br^−^ or I^−^	ND ^b^	ND

^a^ The anions were added in the form of tetra-*n*-butylammonium salts; ^b^ The spectrum changed little and the binding constant could not be calculated.

### 3.4. Interference Experiment

**Figure 3:**
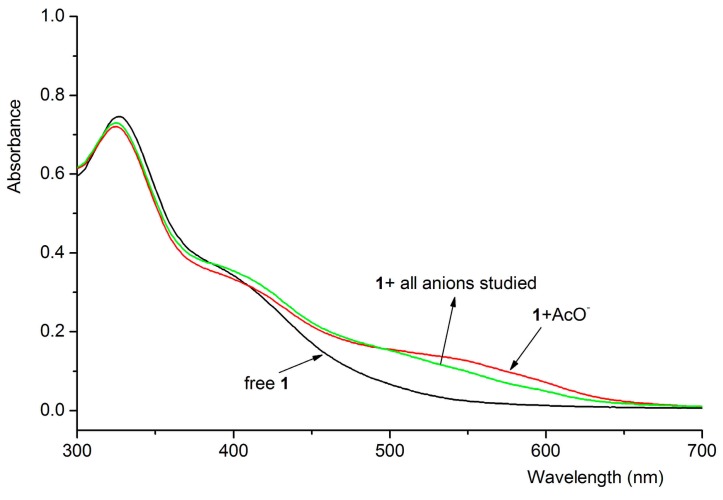
UV-Vis spectral changes of compound **1** (4.0 × 10^−5^ mol·L^−1^) upon the addition of anions (8.0 × 10^−5^ mol·L^−1^).

As shown in Table 1, the binding ability of compound **1** with AcO^−^ ion was the strongest among the anions tested which results from the fact that only one kind of anion existed. Interference experiments were conducted to establish whether the anion binding ability was influenced by the addition of other anions (Figure 3). After various anions (F^−^, AcO^−^ and H_2_PO_4_^−^, 4.0 × 10^−^^5^ mol·L^−1^) were added simultaneously, the spectral responses of compound **1** were different based on the UV-Vis experimental data. As shown in Figure 3, the spectral response of compound **1** upon the addition of anion mixtures involving F^−^, AcO^−^, H_2_PO_4_^−^, Cl^−^, Br^−^ and I^−^ was almost as the same as the addition of AcO^−^ ion. The observation indicated the presence of other anions did not interfere with the binding ability of AcO^−^ ion with compound **1**. Similarly, an identical result was observed in the interaction of compound **3** with AcO^−^. This point has experimental and practical importance. 

### 3.5. Theoretical Investigation

**Figure 4:**
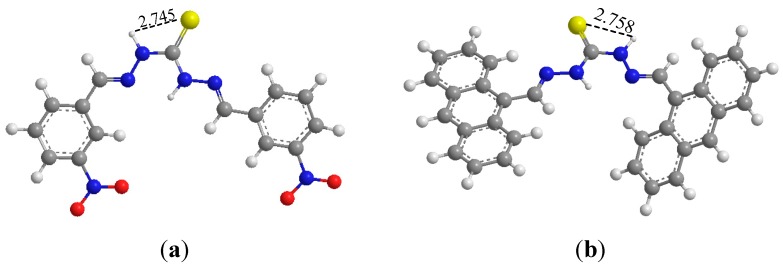
Optimized geometries of (**a**) Compound **1** and (**b**) Compound **3**.

The geometries of **1** and **3** were optimized (Figure 4) using density functional theory at the B3LYP/3-21G level with the Gaussian03 program [37]. As shown in Figure 4, the intramolecular H-bonds between NH and S=C indeed exist in compounds. In compound **1**, the distance between the hydrogen atom and oxygen atom is 2.745 Å, which is close to the distance between two oxygen atoms in AcO^−^ (2.309 Å). Similarly, the distance in compound **3** is 2.758 Å. Therefore, two compounds showed the strongest binding ability for AcO^−^ among the anions tested.

**Figure 5:**
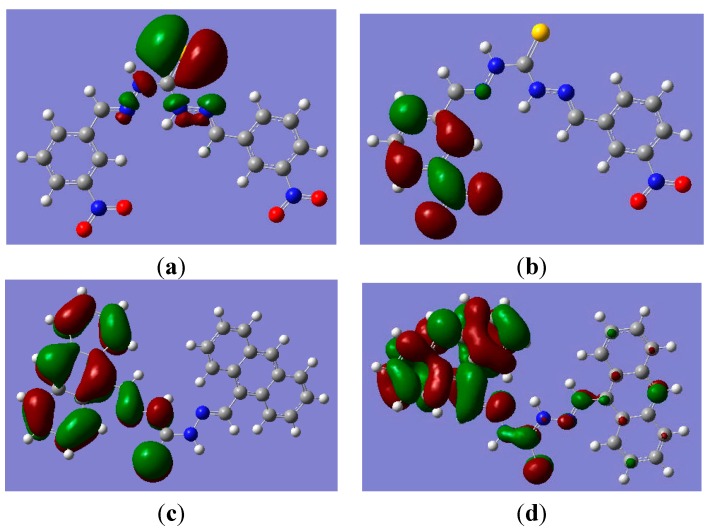
Molecular orbital level of compound **1** and **3**, (**a**) HOMO of **1**; (**b**) LUMO of **1**; (**c**) HOMO of **3**; (**d**) LUMO of **3**.

Selected frontier orbitals for compounds **1** and **3** are shown in Figure 5. The molecular frontier orbitals were introduced in order to explain the red-shift phenomenon in UV-Vis absorption spectra by the electron transitions of frontier orbitals. The highest HOMO density in compound **1** was mainly localized on the interacting thiourea moiety. In contrast, the highest LUMO density was mainly localized on the nitrophenyl moiety, which demonstrated that the electron transition causing the red shift phenomenon in the UV-Vis spectra of **1**-AcO^−^ was from the highest LUMO. For compound **3**, the highest LUMO density was mainly localized on the anthracence moiety, while, the highest HOMO density was localized on the whole molecule, especially on the anthracene moiety. The above results indicated that the red shift phenomenon in the UV-Vis spectra of **3**-AcO^−^ was caused by the electron transition of the highest HOMO density.

## 4. Conclusions

In conclusion, we have developed four symmetrical compounds based on urea/thiourea derivatives. Compound **3** involving thiourea and anthracene groups showed sensitive and selective binding ability for acetate ion through multiple hydrogen bonds in the presence of other competitive anions including F^−^, H_2_PO_4_^−^, Cl^−^, Br^−^ and I^−^ due to the conformational complementarity between the ligand and the analyte and its higher basicity. Compound **3** can be used as a chemosensor for detecting acetate ion in environmental and pharmacy samples. The above results also provide a clue for the construction of anion receptors based on thiourea and anthracene derivatives.
